# The Emergence of Three Human Development Clubs

**DOI:** 10.1371/journal.pone.0057624

**Published:** 2013-03-13

**Authors:** Sebastian Vollmer, Hajo Holzmann, Florian Ketterer, Stephan Klasen, David Canning

**Affiliations:** 1 Department of Economics and Courant Research Center “Poverty, Equity and Growth in Developing Countries”, University of Göttingen, Göttingen, Germany; 2 Department of Global Health and Population, Harvard School of Public Health, Boston, Massachusetts, United States of America; 3 Department of Mathematics and Computer Science, University of Marburg, Marburg, Germany; Cinvestav-Merida, Mexico

## Abstract

We examine the joint distribution of levels of income per capita, life expectancy, and years of schooling across countries in 1960 and in 2000. In 1960 countries were clustered in two groups; a rich, highly educated, high longevity “developed” group and a poor, less educated, high mortality, “underdeveloped” group. By 2000 however we see the emergence of three groups; one underdeveloped group remaining near 1960 levels, a developed group with higher levels of education, income, and health than in 1960, and an intermediate group lying between these two. This finding is consistent with both the ideas of a new “middle income trap” that countries face even if they escape the “low income trap”, as well as the notion that countries which escaped the poverty trap form a temporary “transition regime” along their path to the “developed” group.

## Introduction

There is strong evidence that countries are clustered into at least two groups in terms of income per capita. Quah ([1], [2], [3]) finds evidence of twin peaks in the distribution of income with a cluster of rich countries and a cluster of poor countries. One possible explanation of this clustering into income groups is that countries differ in their underlying characteristics. Bloom, Canning and Sevila [4] reject this hypothesis in favor of a model where countries that are fundamentally the same may either be rich, or may be caught in a self-reinforcing poverty trap from which it is difficult to escape. There is a range of theoretical models consistent with two distinct equilibria and associated poverty traps (e.g. Galor and Zeira [5], Banerjee and Newman [6], Kremer [7]). Whatever the explanation, the fact that there are two “clubs” changes the way we think about economic development. Rather than a continuous process economic growth may require disproportional effort or a “big push” to escape from a poverty trap (Murphy, Shleifer and Vishny [8]).

Similarly, Mayer-Folkes [9] and Bloom and Canning [10] argue that there are two clubs in terms of life expectancy, with one group of countries being clustered around a low level of life expectancy and another being clustered around a high level. Again this is evidence against a smooth progression from low to high life expectancy. We are not aware of similar evidence for education.

In this paper we focus on the *joint* distribution of income per capita, life expectancy and schooling. We focus on these three variables, adding schooling to the established focus on the distribution of income and health, because they have been identified as fundamental determinants of human welfare (e.g. Sen [11]), as reflected, for example, in UNDP”s Human Development Index. In addition to being important for welfare, these three variables are causally interlinked. High income provides resources that can be invested in education and health while health and education are forms of human capital that may lead to high income (e.g. Barro [12], Pritchett and Summers [13]).

We look at the number of clusters in the data graphically using a non-parametric kernel density estimator and also test formally for the number of clusters. We assume that life expectancy, income, and schooling of countries in a cluster have a joint trivariate normal distribution around a common cluster mean. We use a likelihood ratio test for the components in a finite multivariate normal mixture using the parametric bootstrap, which allows for the fact that the distribution of the test statistic in this case is quite non-standard.

We find that in 1960 there are only two clubs in terms of income, health and education. One club has high income, high life expectancy and high education while the other has lower levels of all three variables. By 2000 the picture has changed and we find evidence of three components. We have the same two clubs as before; a high income, high life expectancy and high education club while the other has lower levels of all three variables, though the high income group has advanced in terms of the levels of all three indicators relative to 1960 while the low income, low health and low education has scarcely improved. However we also see the emergence of a third, middle group with income and education levels clustered around a point between those of the two extreme groups but with life expectancy that is only slightly below that of the “developed” club.

Our approach allows us to assign countries to high, middle and low levels of development based on Bayesian posterior probabilities that they are in a group given their observed data on income, health, and education, and therefore we do not have to rely on arbitrary cutoffs to determine group membership. The countries with high probability of membership in the high income, high life expectancy and high education group in 2000 are largely the same as those in this group in 1960. However, the group of countries that had low levels of all three variables in 1960 has split in two, allowing some countries to move up from the low to the middle group.

## Data and Methods

### Data

For income we use GDP per capita, at purchasing power parity, based on 2005 constant prices, calculated using a chain index method. This is the “rgdpch” series from the Penn World Tables Version 7.0 (Heston, Summers and Aten [14]) in log terms. Education is measured using the years of schooling of the population aged 15–64, who are not in school. This is the variable “ty1564” from Cohen and Soto [15]. For health we use life expectancy at birth from the United Nations World Population Prospects: The 2008 Revision (United Nations [16]).

The data on income per capita is annual, while the data on life expectancy is for 5-year intervals. The data on education is available for 1960, 1970, 1980, 1990 and 2000. We average the income and health data to match the education data. For example, we use the average of the GDP per capita observations from 1955 to 1965 as income measure for 1960. We use the average of life expectancy from 1955–1960 and 1960–1965 as health measure for 1960. Our data set includes 84 countries covering about 90% of the world”s population.

### Methods

Gaussian mixture models are often used for cluster analysis, see e.g. Fraley and Raftery [17]. One approach is to choose the number of clusters that best fits the data. Several criteria for goodness of fit have been proposed, including the Bayesian Information Criterion (BIC) and the Integrated Completed Likelihood (ICL) (e.g. Biernacki et al. [18], Fraley and Raftery [17]) and a globally optimal BIC with a potentially restricted covariance matrix (Fraley and Raftery [19]). While we report results for the BIC and ICL selection criteria, our preferred approach is to use a classical testing framework where we test the null hypothesis of 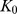 clusters against the alternative of 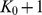 clusters, for each 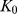, and only reject 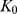 clusters if we reject the null against the alternative at the 5% significance level. This is a conservative approach, which implies that we only accept a larger number of clusters if the data definitely rejects a smaller estimate.

We test for the number of components in the normal mixture models by using the parametric bootstrap. Given data 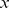 with independent observations 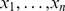, the log-likelihood for a 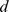-variate Gaussian mixture model with 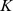 components is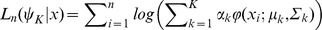 with 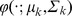 being the density of a 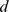-variate normal distribution with mean 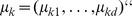 and covariance 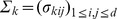 and 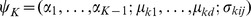) with 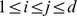 and 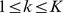.

We use the resampling approach introduced by McLachlan [20] for the assessment of the true null distribution of the likelihood ratio test in testing



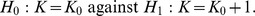



1,000 Bootstrap samples are generated from the mixture model fitted under the null hypothesis of 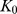 components. That is, the Bootstrap samples are generated from the mixture model with 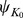 replaced by 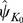, computed by the consideration of the log likelihood formed from the original data under 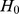. The value of the likelihood ratio teststatistic (LRT) 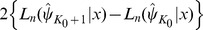 is computed for each Bootstrap sample after fitting mixture models for 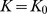 and 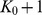 in turn to it. The replicated values of LRT formed from the Bootstrap samples provide an assessment of the Bootstrap and therefore the true null distribution of the LRT. So, the test rejects 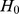 if LRT for the original data is greater than 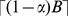 values of LRT for the Bootstrap samples, where 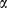 is a prespecified significance level (e.g. 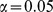).

When determining the number of components, we successively apply this testing procedure for increasing values of 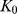 until the hypothesis can no longer be rejected. In order to double-check the conclusions, we also determine the number of components chosen by the model selection criteria BIC (Fraley and Raftery [17]) as well as the ICL (Biernacki et al. [18]).

Once we have fitted a finite mixture model with an appropriate number of components to the data, each observation can be assigned posterior probabilities to belong to each of the components in the mixture model given the data. For a three component normal mixture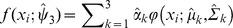 the posterior probability 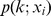 of an observation 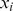 to belong to the 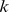
th component is equal to 
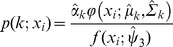
 with 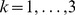. We cluster the data by assigning each observation 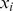 to the component 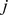 of the mixture to which it has the highest posterior probability of belonging, that is 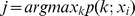.

## Results

**Figure 1:**
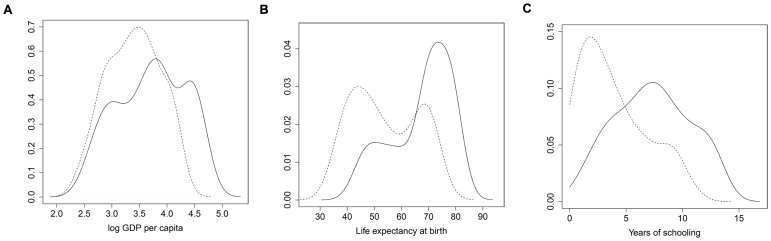
Univeriate distribution of log GDP per capita (base 10), life expectancy and years of schooling in 1960 (dashed line) and 2000 (solid line).

Figure 1 shows a kernel density estimate for the distribution of income per capita in 1960 and 2000. In 1960 we see a unimodal distribution with a single peak. However the distribution does have a “shoulder” to the left, with a mass of low-income countries. If countries are clustered into two groups, and the means of the clusters are far apart, the result will be a twin-peaked distribution. However, if the means of the two clusters are close together the result will be a shoulder in the data as seen in Figure 1 for 1960 income per capita. In general twin-peaked distributions represent at least two clusters (see Vollmer, Holzmann and Schwaiger [21] for a discussion of the relationship between the number of clusters and the number of peaks in the data). By 2000 however we see three peaks in the income per capita distribution.

The graph in Figure 1 for education shows a single peak with a high education shoulder for 1960. For 2000 there is a peak with two shoulders, one above and one below the peak. For life expectancy we see twin peaks in 1960, a tall peak above 40 years and a shorter peak around 70 years. In 2000, we see a single peak around 75 years with a broad shoulder to the left.

We test for the number of normal components in the trivariate mixture distribution. Table 1 shows the bootstrapsed p values (based on 1000 bootstrap repetitions) for the likelihood ratio statistic for one versus two, two versus three, and three versus four components in the distribution for each decade of data. For all decades we reject one versus two components. For 1960 we do not reject two versus three components. It appears that for 1960 the data can be described as a mixture of two trivariate normal distributions. For 1990 and 2000 however, we reject two components against three at the 5 percent level. However, we do not reject three against four components. It appears that we need three components to describe the 1990 and 2000 data. The values of the model selection criteria BIC and ICL, displayed in Tables 2 and 3, confirm these findings. Using restricted covariance models as proposed in Fraley and Raftery [17] supports, after application of appropriate merging algorithms as in Baudry et al. [22], this conclusion so that the result of 3 components in the 2000 data is robust to the use of different methods of identifying these components.

**Table 1:** Bootstrap test for number of components in the trivariate model.

Year	1 vs. 2	2 vs. 3	3 vs. 4
1960	0.00	0.46	0.51
1970	0.00	0.37	0.25
1980	0.00	0.17	0.58
1990	0.00	0.00	0.09
2000	0.00	0.01	0.14

We report the 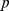-values of a likelihood ratio test with the null hypothesis of 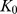 against the alternative of 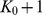 clusters

**Table 2:** BIC and ICL for 1960 and distinct numbers of components.

Number of Components	BIC	ICL
1	1156.98	578.49
2	**1122.91**	**562.79**
3	1144.31	576.44
4	1170.39	590.05

The BIC figures reported are 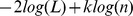, where 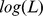 is the log likelihood of the model given the number of components, 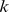 is the number of parameters in the model and 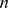 is the sample size. The ICL is the same as the BIC plus a penalty term that increases if the predicted clusters overlap. The best estimate of the number of components is the one with the lowest value of the BIC or ICL.

**Table 3:** BIC and ICL for 2000 and distinct numbers of components.

Number of Components	BIC	ICL
1	1213.219	606.60
2	1171.045	590.73
3	**1162.991**	**582.77**
4	1191.366	596.48

The BIC figures reported are 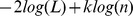, where 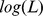 is the log likelihood of the model given the number of components, 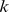 is the number of parameters in the model and 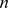 is the sample size. The ICL is the same as the BIC plus a penalty term that increases if the predicted clusters overlap. The best estimate of the number of components is the one with the lowest value of the BIC or ICL.

Tables 4 and 5 show the average characteristics of countries in each cluster assigning each country to the cluster it is most likely a member of (based on the posterior probability). In 2000, the “developed” group has advanced in terms of the levels of all three indicators relative to 1960 while the low income, low health and low education group has scarcely improved; while average education and health levels are slightly higher, income levels are actually substantially lower. We also see the emergence of a third, middle group with income and education levels clustered around a point between those of the two extreme groups but with average life expectancy that is only less than 10% below that of the high level club.

**Table 4:** Descriptive statistics (for 1960).

	Mean	SD	Min.	Max.
	Full Sample (n = 84)			
Years of Schooling	3.9	3.0	0.1	11.0
GDP per capita	4257	4343	259	18955
Life Expectancy at Birth	54.0	12.0	35.0	73.4
	First Component (n = 59)			
Years of Schooling	2.3	1.6	0.1	7.2
GDP per capita	1935	1426	259	6663
Life Expectancy at Birth	47.7	8.1	35.0	68.6
	Second Component (n = 25)			
Years of Schooling	7.5	2.0	3.2	11.0
GDP per capita	9738	3971	4003	18955
Life Expectancy at Birth	69.0	3.0	63.3	73.4

**Table 5:** Descriptive statistics (for 2000).

	Mean	SD	Min.	Max.
	Full Sample (n = 84)			
Years of Schooling	7.3	3.3	0.9	13.1
GDP per capita	11262	12504	369	44834
Life Expectancy at Birth	67.2	11.1	42.7	81.3
	First Component (n = 27)			
Years of Schooling	3.9	2.0	0.9	8.3
GDP per capita	1573	2194	369	11046
Life Expectancy at Birth	52.8	6.1	42.7	62.9
	Second Component (n = 35)			
Years of Schooling	7.5	1.8	3.6	12.3
GDP per capita	6582	3568	2073	18930
Life Expectancy at Birth	71.1	2.6	67.3	77.7
	Third Component (n = 22)			
Years of Schooling	11.3	1.6	7.3	13.1
GDP per capita	30600	6511	17225	44834
Life Expectancy at Birth	78.5	1.1	76.7	81.3

Table 6 shows the posterior probability of each country being in each component of the mixture based on its income, life expectancy and years of schooling. The countries in the high income, high life expectancy, high education group in 2000 are largely the same as those in this group in 1960. Only four countries from the high income, high life expectancy, high education group are part of the middle group in 2000 (Argentina, Jamaica, Trinidad & Tobago and Uruguay). No country from the high income, high life expectancy, high education group in 1960 is part of low income, low health and low education group in 2000.

**Table 6:** Posterior probabilities for each country and component.

Country	1960		2000		
	low	high	low	medium	high
Algeria	1.00	0.00	0.00	1.00	0.00
Argentina	0.05	0.95	0.00	1.00	0.00
Australia	0.00	1.00	0.00	0.00	1.00
Austria	0.00	1.00	0.00	0.00	1.00
Bangladesh	1.00	0.00	0.99	0.01	0.00
Belgium	0.00	1.00	0.00	0.00	1.00
Benin	1.00	0.00	1.00	0.00	0.00
Bolivia	1.00	0.00	0.82	0.18	0.00
Brazil	1.00	0.00	0.00	1.00	0.00
Burkina Faso	1.00	0.00	1.00	0.00	0.00
Burundi	1.00	0.00	1.00	0.00	0.00
Cameroon	1.00	0.00	1.00	0.00	0.00
Canada	0.00	1.00	0.00	0.00	1.00
Central African Republic	1.00	0.00	1.00	0.00	0.00
Chile	1.00	0.00	0.00	1.00	0.00
China	1.00	0.00	0.01	0.99	0.00
Colombia	1.00	0.00	0.00	1.00	0.00
Costa Rica	0.84	0.16	0.00	1.00	0.00
Cote d′Ivoire	1.00	0.00	1.00	0.00	0.00
Cyprus	0.99	0.01	0.00	0.10	0.90
Denmark	0.00	1.00	0.00	0.00	1.00
Dominican Republic	1.00	0.00	0.01	0.99	0.00
Ecuador	1.00	0.00	0.00	1.00	0.00
Egypt	1.00	0.00	0.01	0.99	0.00
El Salvador	1.00	0.00	0.02	0.98	0.00
Ethiopia	1.00	0.00	1.00	0.00	0.00
Fiji	1.00	0.00	0.01	0.99	0.00
Finland	0.01	0.99	0.00	0.00	1.00
France	0.01	0.99	0.00	0.00	1.00
Gabon	1.00	0.00	1.00	0.00	0.00
Ghana	1.00	0.00	1.00	0.00	0.00
Greece	0.03	0.97	0.00	0.02	0.98
Guatemala	1.00	0.00	0.06	0.94	0.00
Haiti	1.00	0.00	1.00	0.00	0.00
Honduras	1.00	0.00	0.01	0.99	0.00
India	1.00	0.00	0.98	0.02	0.00
Indonesia	1.00	0.00	0.02	0.98	0.00
Iran	1.00	0.00	0.03	0.97	0.00
Ireland	0.01	0.99	0.00	0.00	1.00
Italy	0.03	0.97	0.00	0.00	1.00
Jamaica	0.19	0.81	0.00	1.00	0.00
Japan	0.01	0.99	0.00	0.00	1.00
Jordan	1.00	0.00	0.00	1.00	0.00
Kenya	1.00	0.00	1.00	0.00	0.00
Korea, Republic of	1.00	0.00	0.00	0.99	0.01
Madagascar	1.00	0.00	1.00	0.00	0.00
Malawi	1.00	0.00	1.00	0.00	0.00
Malaysia	1.00	0.00	0.00	1.00	0.00
Mali	1.00	0.00	1.00	0.00	0.00
Mauritius	0.98	0.02	0.00	1.00	0.00
Mexico	1.00	0.00	0.00	1.00	0.00
Morocco	1.00	0.00	0.08	0.92	0.00
Mozambique	1.00	0.00	1.00	0.00	0.00
Nepal	1.00	0.00	0.98	0.02	0.00
Netherlands	0.00	1.00	0.00	0.00	1.00
New Zealand	0.00	1.00	0.00	0.01	0.99
Nicaragua	1.00	0.00	0.01	0.99	0.00
Niger	1.00	0.00	1.00	0.00	0.00
Nigeria	1.00	0.00	1.00	0.00	0.00
Norway	0.00	1.00	0.00	0.00	1.00
Panama	1.00	0.00	0.00	1.00	0.00
Paraguay	1.00	0.00	0.00	1.00	0.00
Peru	1.00	0.00	0.00	1.00	0.00
Philippines	1.00	0.00	0.01	0.99	0.00
Portugal	0.13	0.87	0.00	0.12	0.88
Romania	1.00	0.00	0.00	1.00	0.00
Senegal	1.00	0.00	1.00	0.00	0.00
Singapore	0.12	0.88	0.00	0.01	0.99
South Africa	1.00	0.00	1.00	0.00	0.00
Spain	0.03	0.97	0.00	0.00	1.00
Sweden	0.00	1.00	0.00	0.00	1.00
Switzerland	0.00	1.00	0.00	0.00	1.00
Syria	1.00	0.00	0.00	1.00	0.00
Tanzania	1.00	0.00	1.00	0.00	0.00
Thailand	1.00	0.00	0.01	0.99	0.00
Trinidad &Tobago	0.14	0.86	0.02	0.98	0.00
Turkey	1.00	0.00	0.01	0.99	0.00
Uganda	1.00	0.00	1.00	0.00	0.00
United Kingdom	0.00	1.00	0.00	0.00	1.00
United States	0.00	1.00	0.00	0.00	1.00
Uruguay	0.12	0.88	0.00	1.00	0.00
Venezuela	1.00	0.00	0.00	1.00	0.00
Zambia	1.00	0.00	1.00	0.00	0.00
Zimbabwe	1.00	0.00	1.00	0.00	0.00

The group of countries that had low levels of all three variables in 1960 has split in two. Only one country from the low income, low health and low education group in 1960 is part of the “developed” group in 2000 (Cyprus), 27 countries remained in the lowest group, and the other 31 are part of the middle group in 2000. This middle group consists largely of Latin American countries, emerging East Asian economies, and a range of countries from the Middle East.

Those who moved up already started on a higher level of all three indicators in 1960. When examining the development of indicators of this group, one notes a remarkably steady rate of progress in health and education indicators in this group with life expectancy advancing by about 5 years per decade, and education by 1 year per decade. In contrast, GDP growth varies much more (with the 80 s being a particularly low growth period, and the 60 s and 90 s being high growth periods). Those that remained in the poor group developed quite differently over time. After some modest progress in all indicators in the 1960 s and 70 s, income stagnated, and health improvements also slowed down dramatically since then; only education years continued to rise largely unabated. This suggests that these two groups of countries were really on different trajectories leading them to separate into two components. It also suggests that the linkages between the three indicators are not as close as one might surmise. In particular, education improvements seem possible without much income growth and the relation between health and income improvements is also not as close with income fluctuating much more.

**Figure 2:**
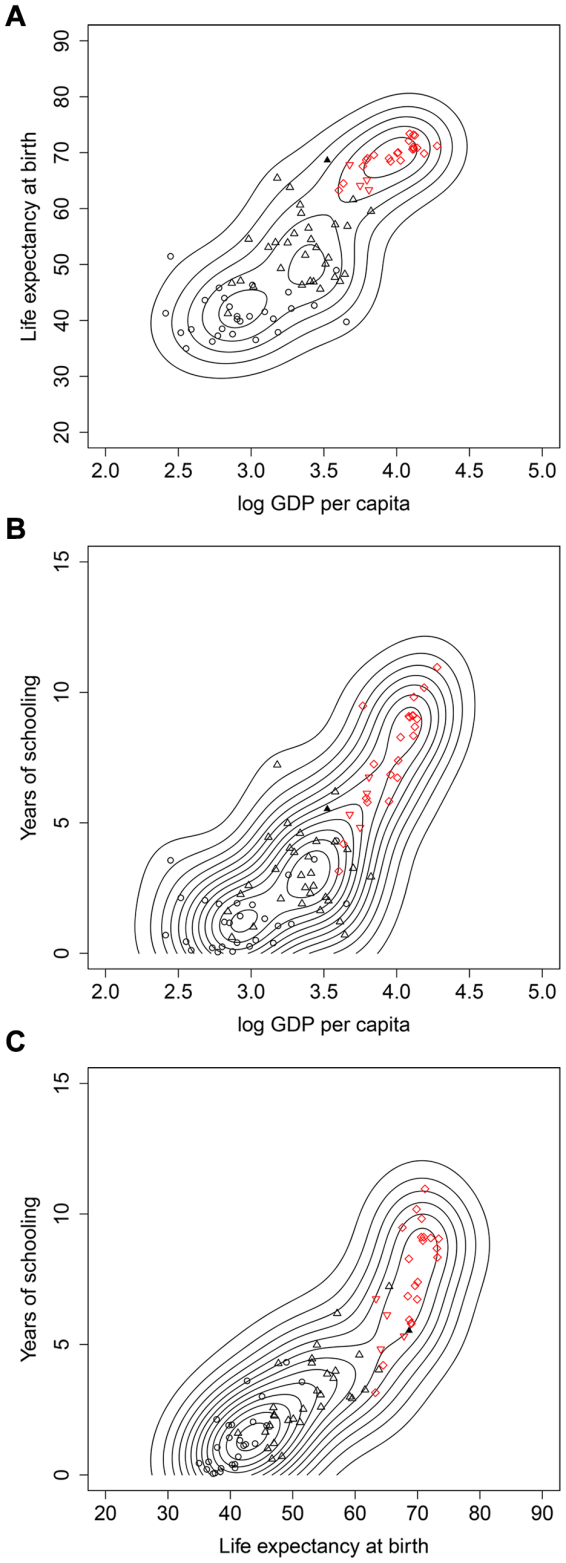
Bivariate distributions of log GDP per capita (base 10), life expectancy and years of schooling in 1960 (black circles: “low” component in both periods, red diamonds: “high” component in both periods component in full three variable model; upward black triangles denote countries from the “low” component that “moved up” and downward red triangles denote countries from the “high” component that “moved down” by 2000).

**Figure 3:**
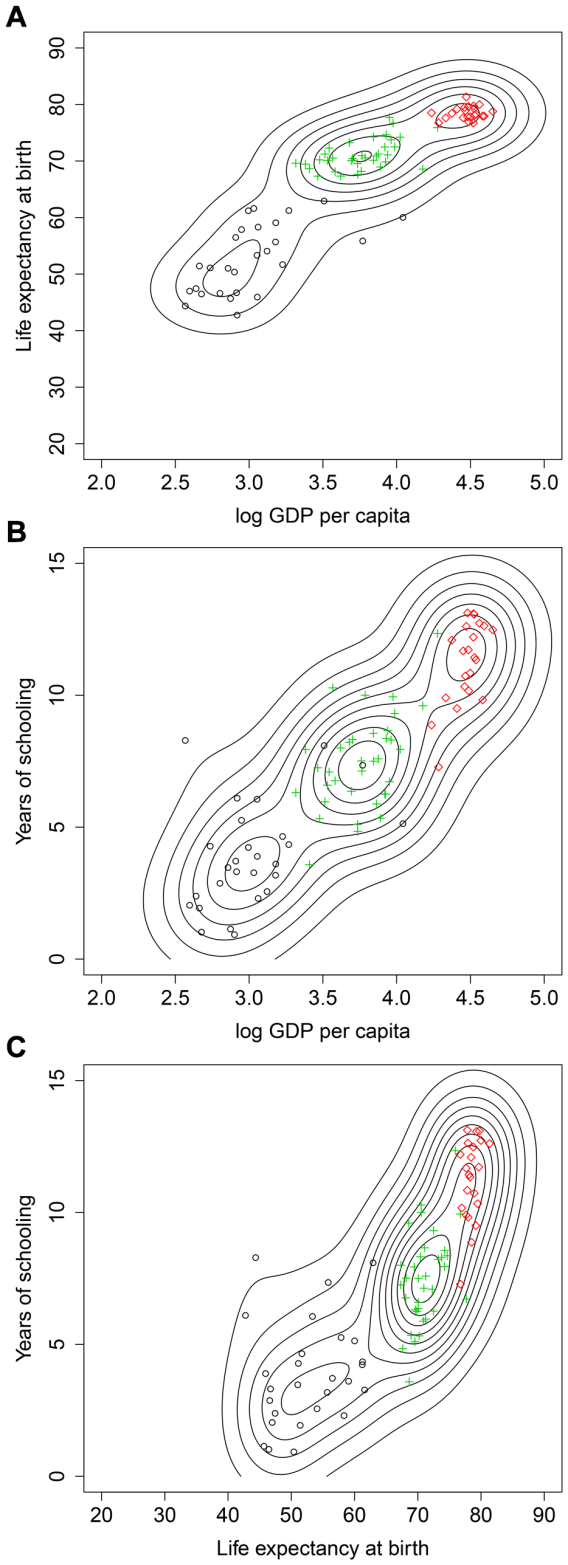
Bivariate distributions of log GDP per capita (base 10), life expectancy and years of schooling in 2000 (black circles: “low” component, green crosses: “middle” component, red diamonds: “high” component in full three variable model).

Figures 2 and 3 contain the contour plots of kernel density estimates for the joint distributions of health with income, education with income, and education with health. The country observations are colored based on their component assignment in the joint trivariate distribution of education, health and income. In Figure 2, countries that leave the “underdeveloped” group by 2000 are symbolized by upward triangles. On average, these countries have higher levels of all three variables in 1960 than countries that stay in the “underdeveloped” group.

## Discussion

We document the emergence of a third development club. In 1960 countries were clustered in two groups; a rich, highly educated, high longevity “developed” club and a poor, less educated, high mortality “underdeveloped” club. By 2000 we see the emergence of three clubs; one underdeveloped group remaining near 1960 levels, a developed group with higher levels of education, income, and health than in 1960, and an intermediate group lying between these two.

This sheds some light on the issue of convergence in development. There is a group of poor countries that are stagnating, and a group of rich countries that are forging ahead, leading to increasing worldwide income inequality. However about half the countries that were poor in 1960 have been very successful, and have seen substantial improvements in income, health and schooling. These countries were already better off in 1960 but were able to steadily enhance income, education and health levels that allowed them to escape from the low development group.

Our results raise the issue of what lies behind the move from a simple “poverty trap” setting in 1960 of two clusters to the three clusters we see in 2000. They emphasize the disparate experience of the underdeveloped countries with one sub-group having done remarkably well while another has largely failed. The emergence of a middle group is consistent with two fundamentally different interpretations. One interpretation could be the idea of a new “middle income trap” that countries face even if they escape the “low income trap” (Griffith [23]); evidence in favor of this view would be the fact that it appears hard to break into the top development group which was achieved by only one country in the sample. Inspection of Figure 3 and Table 5 suggests that the income gap remains massive (with no overlap between the groups) and is not easy to close, particularly in a situation where incomes in the high component also continue rising.

Another interpretation could be the idea that a large number of countries, which escaped the poverty trap, form a temporary “transition regime” along their path to the “developed club” (Galor [24]). If such an interpretation is correct, this implies that the transition does not happen very quickly as only one country moved to the developed club and the gaps (particularly in incomes) remain large. But of course high growth and further rapid improvements in education and health may over time enable the countries of the middle group to transition to the developed group. At this stage, we cannot be sure whether the countries in the middle group will catch up to the “developed club” in the long run or remain in a “middle income trap”.
